# mTOR activity in AIDS-related diffuse large B-cell lymphoma

**DOI:** 10.1371/journal.pone.0170771

**Published:** 2017-02-13

**Authors:** Sara H. Browne, Julio A. Diaz-Perez, Michael Preziosi, Charles C. King, George A. Jones, Sonia Jain, Xiaoying Sun, Erin G. Reid, Scott VandenBerg, Huan-You Wang

**Affiliations:** 1 Division of Infectious Diseases, Department of Medicine, University of California, San Diego, La Jolla, CA, United States of America; 2 Department of Pediatrics, University of California, San Diego, La Jolla, CA, United States of America; 3 Biostatistics Research Center, Department of Family Medicine and Public Health, University of California, San Diego, La Jolla, CA, United States of America; 4 Division of Hematology, Department of Medicine, University of California, San Diego, La Jolla, CA, United States of America; 5 Division of Clinical Pathology, Department of Pathology, University of California, San Diego, La Jolla, CA, United States of America; 6 Human Tissue Technology Core, University of California, San Diego, La Jolla, CA, United States of America; University of North Carolina at Chapel Hill, UNITED STATES

## Abstract

**Background:**

Patients infected with HIV have a significantly increased risk of developing non–Hodgkin lymphomas despite the widespread use of HAART.

To investigate mTOR pathway activity in acquired immunodeficiency syndrome (AIDS) related diffuse large B-cell lymphoma AR-DLBCL, we used immunohistochemistry to examine the presence of the phosphorylated 70 ribosomal S6 protein-kinase (p70S6K), an extensively studied effector of mTOR Complex 1 (mTORC1) and the phosphorylated phosphatase and tensin homolog (pPTEN), a negative regulator of mTORC1 pathway.

**Materials and methods:**

We evaluated tissue samples from 126 patients with AR-DLBCL. Among them, 98 samples were from tissue microarrays (TMAs) supplied by the Aids and Cancer Specimen Resource (ACSR), the remaining 28 samples were from cases diagnosed and treated at the University of California, San Diego (UCSD). The presence of p70S6K was evaluated with two antibodies directed against the combined epitopes Ser235/236 and Ser240/244, respectively; and additional monoclonal anti-bodies were used to identify pPTEN and phosphorylated proline-rich Akt substrate of 40kDa (pPRAS40). The degree of intensity and percentage of cells positive for p70S6K and pPTEN were assessed in all the samples. In addition, a subgroup of 28 patients from UCSD was studied to assess the presence of pPRAS40, an insulin-regulated activator of the mTORC1. The expression of each of these markers was correlated with clinical and histopathologic features.

**Results:**

The majority of the patients evaluated were males (88%); only two cases (1.6%) were older than 65 years of age. We found high levels of both p70S6K-paired epitopes studied, 48% positivity against Ser235/236 (44% in ACSR and 64% in UCSD group), and 86% positivity against Ser240/244 (82% in ACSR and 100% in UCSD group). We observed more positive cells and stronger intensity with epitope Ser240/244 in comparison to Ser235/236 (p<0.0001). The degree of intensity and percentage of cells positive for pPTEN was positively correlated with p70S6K levels (p = 0.016 for 235/236 and p = 0.007 for 240/244). High levels of pPRAS40 were observed in the majority of the cases evaluated (64.3%), but no correlation was found with either pPTEN (p = 0.9) or p70S6K (p = 0.9) levels.

**Conclusion:**

AR-DLBCL frequently contain p70S6K, a main downstream effector of the mTOR pathway. The presence of p70S6K is positively correlated with pPTEN, an inactive form of PTEN, which makes mTORC1 activated. The presence of p70S6K was independent of HIV viral load or CD4 (+) counts. These results suggest that the mTOR pathway is active in the majority of AR-DLBCL, and p70S6K, particularly the Ser240/244 epitope immunohistochemistry is an excellent surrogate biomarker, which could be used to identify cases expected to be responsive to mTOR inhibitors.

## Introduction

Infection with the Human Immunodeficiency Virus (HIV) makes patients more susceptible to viral induced neoplasms including acquired immunodeficiency syndrome (AIDS)-related diffuse large B-cell lymphoma (AR-DLBCL). Lymphomas in these patients often present at advanced stages, frequently with extra-nodal involvement, and have an aggressive clinical course [1]. Co-infection with other viruses such as Epstein-Barr virus (EBV) may contribute to the development of DLBCL [2]. DLBCL is the most common subtype of non-Hodgkin lymphoma seen in HIV infected patients and is associated with EBV co-infection in 30–60% of HIV-infected cases, compared with only 10% in non-HIV cases [3]. Treatment of DLBCL with standard cytotoxic chemotherapy is associated with an increased risk of toxicity, but infusion regimens such as dose-adjusted R-EPOCH appear to be superior to standard R-CHOP chemotherapy in AR-DLBCL [4, 5]. Therefore, advances in rational therapeutic approach are urgently needed. Targeted therapy based on the identification of alterations in signal transduction pathways offers hope of improved efficacy and tolerability [6].

In recent years, the mammalian target of rapamycin (mTOR) has aroused much interest in cancer research. In the cancer setting, the most important control over mTOR activity is via the phosphoinositide-3-kinase/Akt—protein kinase B/mTOR (PI3K/Akt/mTOR) pathway [1, 6]. Two disparate protein complexes have been described: mTOR complex 1 (mTORC1) and mTOR complex 2 (mTORC2). mTORC1 plays a main role in cell proliferation by cell cycle regulation through its downstream effectors 4E-binding protein 1 and 70 ribosomal S6 protein-kinase (70S6K). These effectors initiate translation of mRNA into proteins needed for the cell. mTORC1, in association with raptor, mLST8, and proline-rich Akt substrate of 40kDa (PRAS40), is sensitive to inhibition by rapamycin and its analogs. mTORC2 involved in cytoskeletal dynamics, regulates Akt signaling and does not respond to rapamycin [7]. In addition, the entire mTOR pathway is highly negatively regulated by the phosphatase and tensin homolog gene (PTEN), an upstream tumor suppressor that blocks PI3K signaling when it is not phosphorylated [1, 6].

There is evidence that mTOR is important in other AIDS-related cancers [8–10], and there are several lines of research to suggest that mTOR may be important in AR-DLBCL. In AIDS patients, co-infection with EBV stimulates mTORC1 through mediators such as latent membrane protein 2A and genomic alterations [1, 7]. In the setting of severe immunosuppression and chronic HIV antigenemia, protein synthesis activated by mTORC1 may result in polyclonal and later a monoclonal lymphoproliferation, playing a key role in lymphomogenesis in HIV patients [11]. mTOR inhibitors are effective in inducing cancer cell arrest [8], and some are currently available for low cost, a key factor in treating low-income populations, vital to populations in developing countries where the HIV/AIDS burden is high (72% of all people infected with HIV live in Sub-Saharan Africa) [12].

Available studies have evaluated the overall response rates with mTOR inhibitors at around 30% from DLBCL in immunocompetent patients [13, 14]. Factors behind the responsiveness or resistance to mTOR inhibitors in DLBCL in immunocompetent patients are largely obscure at present and unknown in AR-DLBCL [5, 13]. Several ongoing investigator-initiated trials are evaluating the combination of Everolimus and other mTOR inhibitors in the treatment of DLBCL [1, 5, 6]. The establishment of markers to determine the activity of mTOR in AR- DLBCL and other AIDS related cancers will allow a rational approach to the selection of patients who may further benefit from mTOR inhibition [11].

In order to investigate the extent to which the mTOR pathway is activated in AR-DLBCL, we evaluated the expression of p70S6K with two antibodies directed against the combined epitopes against Ser235/236 and Ser240/244 in 128 patients with AR-DLBCL. In addition, we also investigated the expression of pPTEN, a phosphorylated form of PTEN that activates mTOR pathway.

## Materials and methods

### Population and design

This study was approved by institutional review (The Human Subjects Protection Program of the University of California San Diego (UCSD), IRB #100893, 120802). It met requirements for waiver of informed consent as it posed minimal risk; waiver of consent did not adversely affect the rights or welfare of the subjects; the research could not practicably carried out without the waiver. In addition, a waiver of individual authorization for use of Protected Health Information (PHI) was granted as stipulated by the HIPAA Privacy Rule, 45 CFR 164 section 512(I). We conducted a cross-sectional study: Samples were collected from the UCSD Medical Center (UCSD) (28 cases) and from the Aids and Cancer Specimen Resource (ACSR) (98 cases). The cases from UCSD were diagnosed between 2001 and 2010 in the Division of Hematopathology, Department of Pathology. The ACSR samples were collected in the form of Tissue Microarrays (TMAs) and were supplied by the ACSR to the University of California, San Francisco (UCSF) (for more details, please visit (http://acsr.ucsf.edu/). We analyzed the protein expression of p70S6K and pPTEN on formalin fixed paraffin embedded (FFPE) tissue (conventional sections from UCSD, TMAs from ACSR) by immunohistochemistry (IHC). We then correlated these findings with histopathologic data, and if available, survival data, gender, CD4 count, whether or not subjects were taking antiretroviral medications, and cumulative viral load [15]. Cumulative viral load, which has been linked to all-cause mortality in HIV patients, was calculated for each individual by plotting single viral load measurements as a function of time and taking the area under the curve.

The clinical information on these patients was obtained from review of UCSD medical records. Demographic data were available for ACSR and UCSD as applicable.

### TMAs construction and IHC

The FFPE blocks from the UCSD Medical Center were identified and reviewed following a search in the Department of Pathology database. FFPE tissue blocks were processed by routine methods, and 3 um sections were obtained. Sections were deparaffinized using Histochoice (Sigma-Aldrich) and dehydrated. Dewaxed sections were microwaved for 15 minutes in 1 mM EDTA (pH 8.0), cooled, and treated with 3% H2O2 (Sigma) in 10% methanol to inhibit endogenous peroxidase activity, blocked for 15 minutes at room temperature in solution-B (10% horse serum (Vector Laboratories, Burlingame, CA), 5% BSA, and 0.3% Triton X-100 in PBS) and incubated overnight at 4°C with the appropriate primary antibody: pPTEN, p70S6K (Thr240/Ser244, 1:50), p70S6K (Ser235/236, 1:100), and pPRAS40 (Thr246, C77D7), all of which were purchased from Cell Signaling Technology (Danvers, MA, USA). Following washing, sections were stained for 1 hour with a goat anti–rabbit biotinylated horseradish peroxidase H-conjugated secondary antibody followed by Avidin DH (VECTASTAIN ABC kit; Vector Laboratories). Sections were washed in PBS and were incubated for 5 minutes with Vector *Nova*Red substrate for peroxidase (Vector Laboratories). Slides were counterstained with hematoxylin (Sigma), dehydrated in 95% alcohol and absolute alcohol, cleared in xylene, and mounted in Permount (Sigma). New H&E slides for each case were also made.

The ACSR provided unstained TMAs made from FFPE tissue block samples obtained from representative areas (rich in lymphoma cells). A minimum of two cores per patient with a diameter of 2 mm was obtained from different areas of each block. Other tissues cores (38 samples) were included as controls (29 reactive lymph nodes and 9 non AR-DLBCL) to make a total of 135 cores. The IHC in TMAs was performed the same as insamples from the UCSD Medical Center.

### Semi-quantification of IHC and histopathologic findings

The results of the IHC were evaluated and scored by two hematopathologists (HYW and JDP) independently, who were also blinded to clinical information. The degree of intensity and percentage of positivity were separately recorded, and the IHC score is semi-quantified, defined as result of degree of intensity multiplying the percentage of positive cells. Positive scores were defined as an IHC score of 2 or greater. The score for degree of staining intensity was graded from 0 to 2 (0 as null, 1 as dim and 2 as strong); the score for the percentage of positive cells was graded from 0 to 3 (0 = null, 1 = 1–9%, 2 = 10–49%, or 3 = 50–100%). The cutoff for positivity was set at 10% of tumor cells staining with mTOR signaling-related antibodies. All tumor samples assessed as negative to mTOR signaling related antibodies were assessed for the presence of stromal staining by these antibodies providing a positive internal control.of tissue integrity and quality of staining.

Histopathologic features of the AR-DLBCLs including cytology and histology of tumor cells, non-tumor cell types, and presence or absence of necrosis were evaluated. Images were recorded using a LEICA DM LA histology microscope (Leica, Heidelberg, Germany) equipped with a camera.

### Genomic analysis

#### Extraction of RNA from tissue and PCR array

Total RNA was extracted from FFPE sections using the RNeasy FFPE Kit as described by the manufacturer (Qiagen, Valencia, CA). From this, mRNA was isolated using the RNeasy Kit (Qiagen). Due to low amounts of extracted mRNA, the RT2PreAMP cDNA Synthesis Kit (Qiagen) was used to preamplify an array-specific set of targets prior to using an RT^2^ Profiler PCR Array. Amplified cDNA was assayed using either the human HIV Host Response PCR Array or the mTOR Signaling PCR Array. Data was analyzed using the RT^2^ Profiler PCR Array for Data Analysis (http://pcrdataanalysis.sabiosciences.com/pcr/arrayanalysis.php) [16].

#### Statistical analysis

Descriptive analyses were performed on the demographics, clinical and histopathological features of the samples. IHC scores were summarized overall and by morphological classification and organ types. Wilcoxon Rank Sum test was used for comparison between the groups. Pairwise Spearman’s rank correlations were calculated among the IHC scores, as well as between the IHC scores and clinical features including cumulative viral load, nadir CD4, CD4 at biopsy, and Ki-67. Cumulative viral load, which has been linked to all-cause mortality in HIV patients, was calculated for each individual by plotting single viral load measurements as a function of time and taking the area under the curve.

Analyses were performed separately for the two samples (TMA and UCSD). A p-value of <0.05 was considered statistically significant. Statistical analyses were performed in R (http://cran.r-project.org), version 3.0.2.

## Results

### Demographics in AR-DLBCL

Of the TMA samples in the ACSR, 86% (84/98) were male, 12% (12/98) female, and 2 unknown. In the UCSD cases, 96% (27/28) were male and 4% (1/28) female. The mean age for both groups was similar: 42 years (range from 22 to 66 years) and 44.4 years (range from 24 to 68 years) for the ACSR and the UCSD patients, respectively (Table 1). Of the total 126 patients, only 1.6% (2/126) were older than 65 years of age.

**Table 1:** Clinical characteristics and histopathologic features of AR-DLBCL cases.

Characteristic		ACSR study group	UCSD study group
Median Age (Range) (years)		42 (22–66)	45 (24–68)
Sex			
	Females	12(12.2%)	1 (3.6%)
	Males	84(86.5%)	27 (96.4%)
Mean CD4+ cells nadir (Range)		Not available	81.2 (5–327)
CD4+ cells count at biopsy time		Not available	212.3 (9–595)
Cumulative HIV viral load		Not available	16.9 (0.66–43.64)
Site of biopsy			
	Extra-nodal	46 (46.9%)	17 (60.7%)
	Nodal	39 (39.8%)	10 (35.7%)
	No identified	13 (13.3%)	1 (3.6%)
Morphological Classification			
	Centroblastic	66 (67.3%)	20 (71.4%)
	Immunoblastic	4 (4.1%)	0 (0.0%)
	Anaplastic	0 (0%)	1 (3.6%)
	Not defined	28 (28.6%)	7 (25.0%)
Architecture			
	Diffuse	97 (98.9%)	28 (100%)
	Nodular	1 (1.0%)	0 (0%)
Necrosis			
	Present	83 (84.7%)	6 (21.4%)
	Absent	15 (15.3%)	22 (78.6%)
Ki-67 positivity (mean) (range)		63.47% (5–100%)	73.42% (5–100%)
EBV positivity		40 (40.8%)	2 (50%)^1^

**Abbreviations:** ACSR: AIDS and Cancer Specimen Resource. UCSD: University of California, San Diego.

EBV: Epstein-Bar virus.

^1^Among the 28 AR-DLBCLs, EBV immunohistochemistry was done only in 4 cases, 2 of which were positive for EBV.

### ACSR study group

#### Clinical and histopathologic features

The biopsy site for these AR-DLBCLs from high to low was as follows: lymph nodes (39 cases), gastrointestinal tract (12 cases), mouth (5 cases), skin (3 cases), brain (3 cases), testis (3 cases), and other locations. Duplicate samples from 98 AR-DLBCL were analyzed in TMAs. 36.7% (36/98) were classified according to cell of origin as DLBCL with germinal center (GC) phenotype according to published criteria [17], 40.8% (40/98) were classified as having EBV expression. Limited available clinical data on these cases were as follows: history of standard antiretroviral therapy was documented in 31.6% (30/98) of cases. 8 additional cases had a history of antiviral therapy with acyclovir and analogs; 5 cases were treated previously with radiotherapy and 24 cases received different chemotherapy regimens, details of which were not available.

In all samples, lymphoma cells accounted for >80% of all cells. These lymphomas were predominantly polymorphic (93 cases) and all contained from 10 to 90% neoplastic centroblasts (1A). Hodgkin-like cells were also observed in 11 cases (11.5%), immunoblasts in 13 cases (13.5%) and the latter were predominant in 4 cases. These lymphomas mainly showed a diffuse growth pattern 99% (97/98) with only 1 case of nodular morphology. Necrosis was evident in 15.3% (15/98) cases. The predominant tumor microenvironment cell was most commonly small mature lymphocytes (78 cases), followed by histiocytes (15 cases) and eosinophils (2 cases).

#### 70S6K is frequently phosphorylated in AR-DLBCL

**Fig 1:**
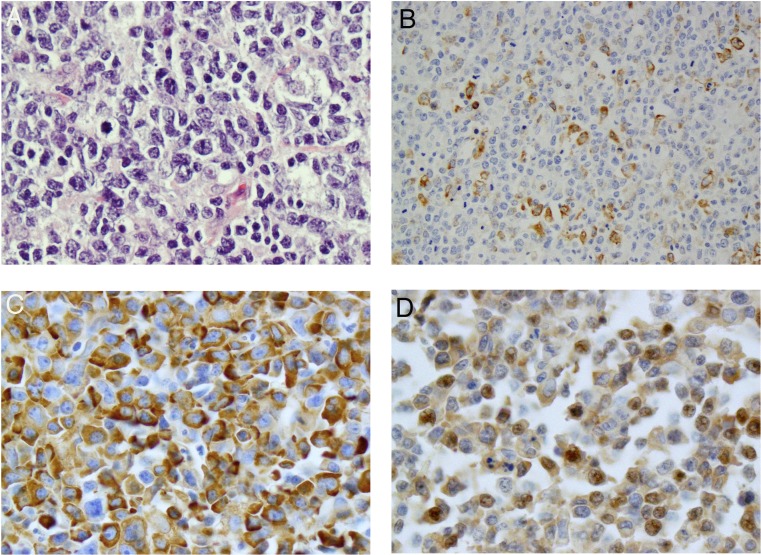
Morphologic and immunohistochemical features of AR-DLBCL. **A**: A representative of the AR-DLBCL cases show centroblast morphology (H&E, original magnification 400X); **B**: A representative case of AR-DLBCL with approximately 10% of the lymphomatous cells positive for p70S6K 240/244 (original magnification 200X); C: A representative case of AR-DLBCL with approximately 90% of the lymphomatous cells with strong immunoreactivity for p70S6K 240/244 (original magnification 400X); D: A representative case of AR-DLBCL with the majority of the lymphomatous cells showing moderate nuclear immunoreactivity for pPTEN (original magnification 400X).

An antigen is considered positive if equal or more than 10% of tumor cells are positively stained compared to the background non-stained cells (Fig 1B). The positive stain for p70S6K-235/236 in 44% cases (43/98) and for p70S6K-240/244 (Fig 1C) in 82% cases (80/98) (Table 2) was observed. Mean IHC score for the p70S6K-240/244 was significantly higher than that of the p70S6K-235/236 (4.21 vs. 2.03; *p*<0.001), although the two scores were highly associated (r = 0.76; p<0.001). AR-DLBCL with an IHC score of ≥4 for p70S6K-240/244 were observed in 79% cases (77/98) compared to 29% (28/98) cases for p70S6K-235/236. p70S6K-240/244 score was higher EBV+ DLBCL in comparison to DLBCL without evidence of EBV, with a median score = 6 for EBV+ versus a median score = 4 for other DLBCL, p-value = 0.083. While the p value shows a trend, it did not reach significance, possibly due to the small sample size.

**Table 2:** Phosphorylation profile by IHC.

	Positive samples = Number of samples in which >10% of cells taking up stain	Mean IHC score integrating percent of cells taking up stain and intensity (0–6)
***ACSR (Training) Group N = 98***		
p70S6K epitope 235/236	43.9% (43/98)	2.03
p70S6K epitope 240/244	81.6% (80/98))	4.21
pPTEN	76.5% (75/98)	3.37
***UCSD Study Group N = 28***		
p70S6K epitope 235/236	64.3% (18/28)	2.18
p70S6K epitope 240/244	100% (28/28)	3.61
pPTEN	50.0% (14/28)	2.75
pPRAS40	64.3% (18/28)	2.56

PTEN is phosphorylated in majority of AR- DLBCL and correlates with 70S6K phosphorylation.

Seventy seven percent of cases (75/98) were positive for pPTEN (Fig 1D), the main negative regulator of the mTOR (Table 2); phosphorylation is indicative of PTEN inactivation. Average IHC score was 3.37; 63% of samples testing positive had IHC scores ≥4. IHC scores for pPTEN were positively correlated with that of p70S6K 240/244 (r = 0.27, *p* = 0.007) and p70S6K 235/236 (r = 0.24, *p* = 0.016).

This correlation was stronger in the cases that were morphologically subtyped as GCB-DLBCL (p70S6K 240/244 r = 0.35, *p* = 0.035; 235/236 r = 0.32, *p* = 0.059).

### UCSD study group

#### Clinical and histopathologic features

Twenty-eight samples from the same number of patients were identified from UCSD, along with their clinical information including viral load, cumulative viral load, CD4 count (nadir and at time of biopsy), and response to chemotherapy with regard to complete response (CR), partial response (PR), stable disease (SD), progression of disease (PD), and overall survival (OS).

The biopsy site in descending order was lymph nodes (10 cases), gastro-intestinal tract (6 cases), brain (3 cases), bone marrow (2 cases), soft tissues (2 cases), lung (1 case), skin (1 case), testis (1 case) and uterus (1 case) (Table 1). All patients received antiretroviral therapy. With respect to HIV risk factors, the patients were predominantly men that have sex with men (42.8%), followed by intra-venous drugs users (17.9%) and heterosexuals (10.7%), in 8 cases the past history was unknown.

In all samples evaluated, lymphoma cells accounted for >90% of total cells. All lymphomas (100%, 28/28) showed polymorphic cell infiltrate comprised of large neoplastic lymphoid cells distributed in a diffuse pattern. 71.4% (20/28) were classified as having centroblastic morphology, and one as having anaplastic morphology (4.2%). Twenty one percent (6/28) showed tumor necrosis. The predominant microenvironment cell was most commonly lymphocytes (in 21 cases), followed by macrophages (4 cases), eosinophils (2 cases) and neutrophils (1 case).

#### 70S6K is frequently phosphorylated in AR-DLBCL

Positive stain for p70S6K-235/236 in 64.3% (18/28) and 100% (28/28) for p70S6K-240/244 (Table 2) was observed, respectively. Mean IHC score was 3.61 for the p70S6K-240/244 and 2.18 for the p70S6K-235/236, respectively, similar to the TMA samples. An IHC score of ≥4 was found for p70S6K-240/244 in 39.3% (11/28) and for p70S6K-235/236 in 21.4% (6/28), respectively.

#### PTEN and PRAS40 are phosphorylated in the majority of AR-DLBCL

Expression of pPTEN, the hyperphosphorylated form of PTEN, was seen in 50% (14/28) of the cases (Table 2). Average pPTEN IHC score was 2.75. There was no significant difference in pPTEN expression between GCB versus non-GC subtypes or Ki-67 expression. 64.2% cases (18/28) were positive for pPRAS40 (Table 2), a main activator of mTOR and marker of Akt activation. Average IHC score for pPRAS40 was 2.56. In this small cohort, no significant correlation between pPTEN IHC scores and those of the p70S6K 240/244 epitope was found, (r = 0.16, p = 0.43). pPRAS40 IHC scores were not correlated with those of either p70S6K 240/244 epitope (r = 0.03, *p* = 0.87) or pPTEN (r = 0.03, *p =* 0.90).

#### 70S6K phosphorylation is not correlated to cumulative HIV viral load or CD4 count

No significant correlation between cumulative viral load and IHC score [p70S6K-240/244 (r = 0.18, p = 0.38); pPTEN (r = 0.21, p = 0.29); pPRAS-40 (r = -0.15, p = 0.48)) was found. Similarly, no association was observed between cumulative viral load and CD4 count, either at time of biopsy or at nadir. There was a moderate correlation between p70S6K scores with the expression of Ki-67[p70S6K-235/236 (r = 0.44, p = 0.06), p70S6K240/244 (r = 0.19, p = 0.44)], but not pPTEN (r = 0.009 p = 0.97), or pPRAS40 (r = -0.093, p = 0.7). We further examined the potential associations between 70S6K, PTEN and PRAS40 phosphorylation and clinical features including nadir CD4 count, CD4 count at time of biopsy and response to chemotherapy.

No significant associations were found. Finally, when a univariate analysis of associations between 70S6K epitope phosphorylation, viral load (VL), CD4 nadir, and CD4 count at time of biopsy with OS was performed, only CD4 count both at the time of biopsy and CD4 nadir were significantly associated with OS [(r = 0.7, P = 0.02) and (r = 0.73. P = 0.05)], respectively.

#### Gene expression comparison

In order to investigate if there is an alteration of gene expression in association with elevated mTOR signaling, gene expression profiles using the mRNA extracted from AR-DLBCL were performed. Due to technical difficulties of working with older formalin fixed samples, we were only able to extract mRNA from one patient tissue sample with activated mTOR and one patient tissue sample with minimal mTOR activity based on the IHC. While the examined samples are very limited, the results are very dramatic, namely, there is a uniform 3- to 6-fold increase in expression of the mTOR-related genes in mTOR activated case compared to minimally activated case (S1 Table). These results should be interpreted with caution due to the small number of cases, however we have included these as this data is rarely obtained and did suggest that the gene expression profile in these samples is similar to their IHC findings regarding mTOR signaling. A larger sample pool will help us identify critical genes in this pathway and look at their corresponding IHC findings.

## Discussion

In our study of the PI3K/Akt/mTOR pathway, effector proteins represented by 70S6K, PRAS40, and PTEN are shown to be phosphoylated in the majority of AR-DLBCL cases. At the protein level, in the case of downstream effectors 70S6K and PRAS40, this activation is manifested by the high number of cells and enhanced intensity observed of anti-p70S6K antibody for both paired epitopes (Ser235/236) and (Ser240/244) by IHC.

In addition, the IHC demonstrated that the anti-p70S6K 240/244 antibody showed much higher levels than the anti-p70S6K-235/236 antibody indicating the 240/244 240/244 epitope is a superior surrogate marker for activation of this pathway in AR-DLBCL with 82–100% positivity in AR-DLBCL. In the case of upstream regulator PTEN, phosphorylation was also observed in the majority of AR-DLBCL, again consistent with the relief of the negative suppression on the PI3K/AKT/mTOR signaling pathway by PTEN [18].

Our results are in agreement with those of El-Salem et al. who identified p70S6K activation in the majority of 24 cases of AR-DLBCL, although they did not report the exact percentage of positive cases [7]. However, in contrast to Sebestyen et al [11] who reported 80% p70S6K immunoreactivity in DLBCL with non-GC phenotype but none in DLBCL with GC phenotype, our exclusively AR-DLBCL sample showed similar p70S6K immunoreactivity between GC and non-GC types.

In agreement with the phosphorylation of 70S6K in the majority of the AR-DLBCL cases, the phosphorylation of PTEN, a tumor suppressor and the major negative regulator of the PI3K/AKT/mTOR pathway, was observed in 68–77% of AR-DLBCLs, substantiating our findings that the negative suppression of mTOR in AR-DLBCL was relieved by PTEN. Of interest, non-AR-DLBCL also has similar PTEN findings. However, in non-AR-DLBCL, Pfeifer et al. report 47% of GCB DLBCLs stained for PTEN in comparison to 87% in non-GCB DLBCLs leading to a proposal of PTEN loss that defines a PI3K/AKT dependent pathway in GCB DLBCL [19]. In our AR-DLBCL training samples from ACSR, pPTEN was positively correlated with p70S6K expression, but there is no significant difference in term of pPTEN expression between GCB and non-GCB. While the mechanism for such discrepancy is not clear, the DLBCLs studied in our experiment are exclusively AR-DLBCL, in contrast to those from Pfeifer et al [19]. PRAS40 was phosphorylated in 64% of AR-DLBC from UCSD samples. pPRAS40 is both a substrate of Akt and a component of the mTORC1 complex. High levels of activated Akt have been shown to correlate with a deteriorating clinical course in non-AR-DLBCL [20].

The AR-DLBCLs from UCSD patients were associated with extensive clinical data, which were unfortunately not available from the ACSR samples. In AR-DBCLs from UCSD, we did not observe a significant correlation between cumulative viral load and 70S6K phosphorylation. Similarly, no association was observed between 70S6K phosphorylation and CD4 count, either at time of biopsy or at nadir. While there could be no true association between 70S6K phosphorylation and viral load as well as CD4 count, the small sample size could be the real reason. In this small cohort, OS was only associated with CD4 count at time of biopsy and CD4 nadir, consistent with the published literature [21].

The majority of studies of the mTOR signal pathway in non-Hodgkin lymphomas have been performed on cell lines [14, 22]. In these preclinical studies, rapamycin or its analogs have been demonstrated to have a significant anti-proliferative effect [8, 22], and influence angiogenesis [23, 24]. mTOR related proteins and inhibitors are clearly promising as prognostic biomarkers and therapeutic targets in DLBCL [25]. Previous results in non-HIV/AIDS populations have demonstrated that DLBCL patients with active mTOR pathway have a trend toward shorter OS when compared to patients with mTOR negative tumors [11]. A clinical trial by Witzig et al. to evaluate mTOR inhibitors has shown modest responses (objective overall response rate = 30%) [14]. However, the patients were highly compromised by multiple comorbidities and were not selected on the basis of tissue identification of mTOR activation. We support the use of limited immunohistochemical markers to identify potential mTOR inhibitor therapy candidates in accordance with previous studies [11, 26–28]. Thus, finding an overall response rate of 30% is meaningful and provides the rationale for further studies evaluating the use of mTOR as adjuvant therapy in AIDS related lymphoma, which is frequently very aggressive.

In conclusion, we show that AR-DLBCL exhibits high levels of mTOR expression. Immunohistochemistry of the surrogate marker, p70S6K and pPTEN, could potentially be used to predict activation of mTOR, and identify cases potentially responsive to rapamycin and its analogs as adjuvant therapy.

## Supporting information

S1 Table Gene expression comparison between a pKS6 negative (sample 1) and a pKS6 positive (sample 2) cases. (DOCX)

S1 File TMA DLBCL Data Set. (ZIP)
