# Supplementary material for: mTOR activity in AIDS-related diffuse large B-cell lymphoma
Source: PLoS One. 2017 Feb 13;12(2):e0170771. doi: 10.1371/journal.pone.0170771 (PMC5305194; doi:10.1371/journal.pone.0170771)
Supplement: S1 Table — (DOCX) [file pone.0170771.s001.docx]

**S1 Table. Gene expression comparison between a pKS6 negative (sample 1) and a pKS6 positive (sample 2) cases.**

| **Gene** | **Sample 1** | **Sample 2** | **2^-ΔCt Sample 1** | **2^-ΔCt Sample 2** | **Fold Change** |
| --- | --- | --- | --- | --- | --- |
| EIF4B | -0.02 | 9.84 | 1.00E+00 | 1.10E-03 | 933.67 |
| PRKCE | 2.047834843 | 5.066522385 | 0.241846767 | 0.029841784 | 8.104299791 |
| PRKAG3 | 7.00173813 | 9.842486779 | 0.007803093 | 0.001089222 | 7.163917131 |
| AKT1 | 7.235569357 | 9.842486779 | 0.006635545 | 0.001089222 | 6.092006251 |
| CAB39L | 7.235569357 | 9.842486779 | 0.006635545 | 0.001089222 | 6.092006251 |
| DDIT4L | 7.235569357 | 9.842486779 | 0.006635545 | 0.001089222 | 6.092006251 |
| INS | 7.235569357 | 9.842486779 | 0.006635545 | 0.001089222 | 6.092006251 |
| INSR | 7.235569357 | 9.842486779 | 0.006635545 | 0.001089222 | 6.092006251 |
| IRS1 | 7.235569357 | 9.842486779 | 0.006635545 | 0.001089222 | 6.092006251 |
| PPP2R2B | 7.235569357 | 9.842486779 | 0.006635545 | 0.001089222 | 6.092006251 |
| PRKCG | 7.235569357 | 9.842486779 | 0.006635545 | 0.001089222 | 6.092006251 |
| TELO2 | 7.235569357 | 9.842486779 | 0.006635545 | 0.001089222 | 6.092006251 |
| TSC2 | 7.235569357 | 9.842486779 | 0.006635545 | 0.001089222 | 6.092006251 |
| VEGFC | 7.235569357 | 9.842486779 | 0.006635545 | 0.001089222 | 6.092006251 |
| PIK3CG | 7.235569357 | 9.786469023 | 0.006635545 | 0.001132346 | 5.859995944 |
| ULK1 | 6.092723198 | 8.313277511 | 0.014652359 | 0.003143794 | 4.660724748 |
| PRKAB2 | 3.234891858 | 5.279918125 | 0.106218587 | 0.02573868 | 4.126807851 |
| RPS6KA2 | 5.833776214 | 7.869608121 | 0.017533086 | 0.004275746 | 4.100591152 |
| RPTOR | 4.818544346 | 6.805778755 | 0.03543836 | 0.008938331 | 3.964762393 |
| MAPK3 | 6.037155118 | 7.936474941 | 0.015227732 | 0.004082094 | 3.73037282 |
| HPRT1 | 1.768937676 | 3.569133445 | 0.29342472 | 0.08425269 | 3.482674808 |
| PRKAG2 | 7.235569357 | 9.014356974 | 0.006635545 | 0.001933785 | 3.431376944 |
| PRKAA2 | 7.235569357 | 8.993460962 | 0.006635545 | 0.001961998 | 3.382035035 |
| RPS6 | -6.193027103 | -4.505818518 | 73.1622279 | 22.71885966 | 3.220330113 |
| RICTOR | 7.235569357 | 8.888552257 | 0.006635545 | 0.002109984 | 3.144831894 |
| STK11 | 2.853565509 | 4.467735477 | 0.13835383 | 0.04519367 | 3.061354186 |
